# Supplementary material for: DMRforPairs: identifying Differentially Methylated Regions between unique samples using array based methylation profiles
Source: BMC Bioinformatics. 2014 May 15;15:141. doi: 10.1186/1471-2105-15-141 (PMC4046028; doi:10.1186/1471-2105-15-141)
Supplement: Additional file 2 — DMRforPairs output for the comparison of A431-MCF7 and NA17018-NA17105. Please start from the HTML files in each folder. Available via the BMC Bioinformatics website. [file 1471-2105-15-141-S2.zip › 1394847754114233_MOESM2_ESM/A431_MCF7/figures/6943.pdf]

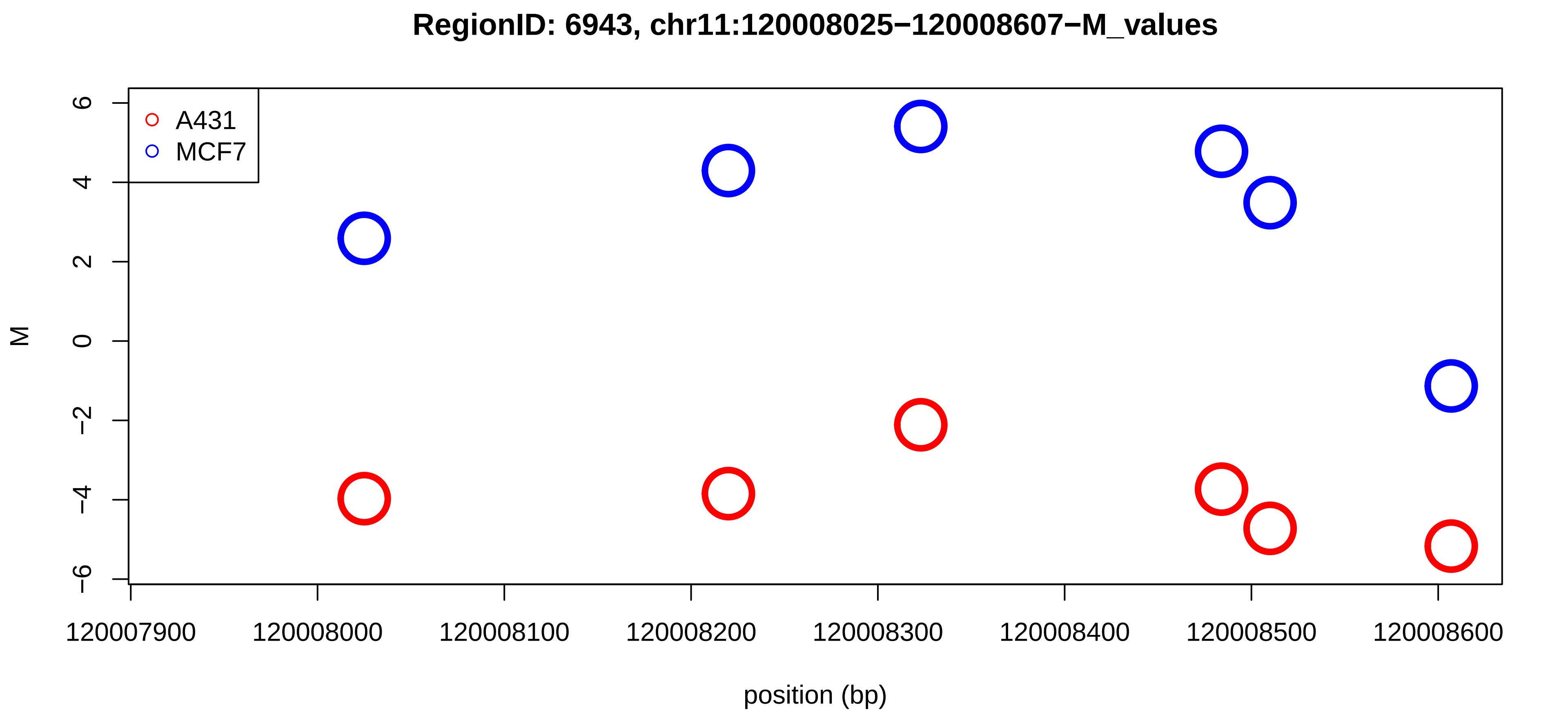
RegionID: 6943, chr11:120008025−120008607−M_values
A431
MCF7
M
6
4
2
0
−2
−4
−6
120007900
120008000
120008100
120008200
120008300
120008400
120008500
120008600
position (bp)

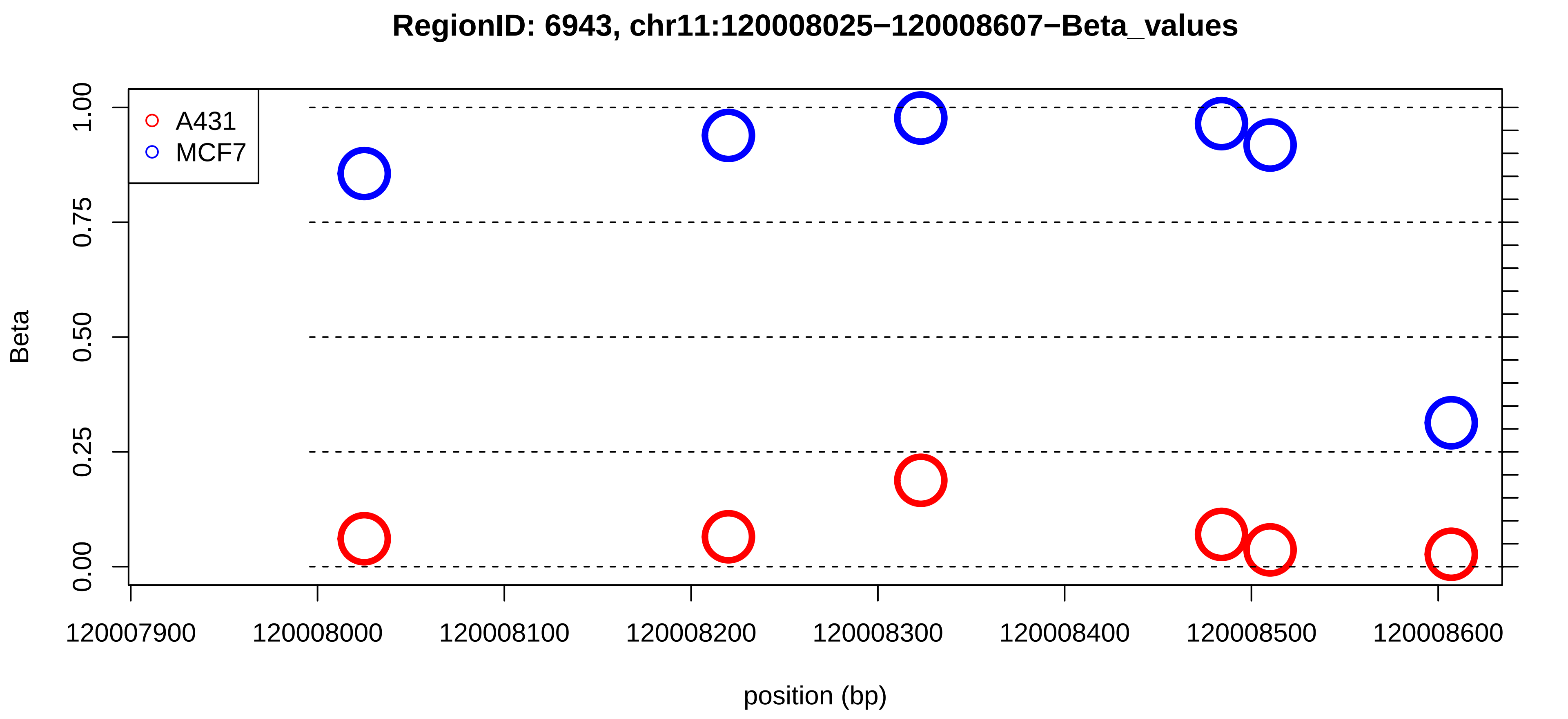
RegionID: 6943, chr11:120008025−120008607−Beta_values
A431
MCF7
Beta
1.00
0.75
0.50
0.25
0.00
120007900
120008000
120008100
120008200
120008300
120008400
120008500
120008600
position (bp)

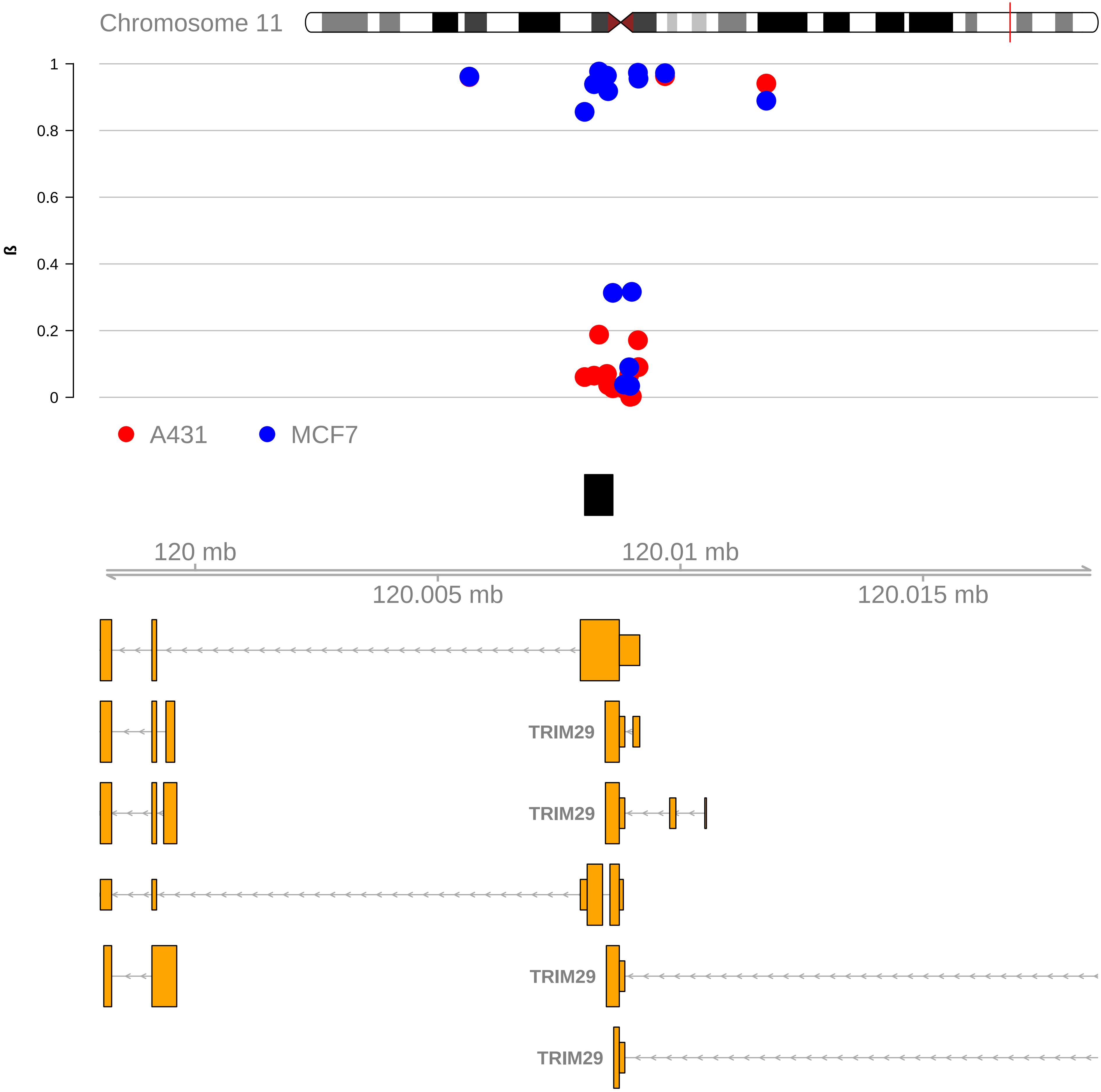

Chromosome 11
1
0.8
0.6
0.4
0.2
0
β
A431
MCF7
120 mb
120.01 mb
120.005 mb
120.015 mb
TRIM29
TRIM29
TRIM29
TRIM29
